# Thrombosis of the Portal Vein and Superior Mesenteric Vein in a Patient With Subclinical COVID-19 Infection

**DOI:** 10.7759/cureus.14366

**Published:** 2021-04-08

**Authors:** Jasneet Randhawa, Jasveen Kaur, Harneet S Randhawa, Sifatpreet Kaur, Harinderpal Singh

**Affiliations:** 1 Medical Officer, Fortis Escorts Hospital, Amritsar, IND; 2 Medical Officer, National Health Mission, Amritsar, IND; 3 Radiodiagnosis, Government Medical College, Baramati, IND; 4 Anaesthesiology, Government Medical College, Chandigarh, IND; 5 Non-Invasive Cardiology, Fortis Escorts Hospital, Amritsar, IND

**Keywords:** sars-cov-2, portal thrombosis, superior mesenteric vein thrombosis, covid coagulopathy, sars-cov-2 testing, covid-19 antibody positivity rate, covid-19 symptoms, asymptomatic covid-19, clinical diagnosis of covid-19, covid complications

## Abstract

More than 122 million cases of COVID-19 infection have been documented, and hundreds of thousands are being added every day. Several co-morbidities are associated with COVID-19, among which hypercoagulability has garnered the attention of many doctors and researchers.

Most cases of vascular thrombosis are noted in intensive care unit (ICU) patients with serious disease; among these, many cases of deep venous thrombosis and pulmonary embolism have been noted. A few cases of portal vein thrombosis have also been documented in ICU patients with severe COVID-19.

Here, we present a case of a portal vein and superior mesenteric vein thrombosis in a patient with subclinical COVID-19 infection. Through this case report, we intend to increase the research horizon and wish to help diagnose co-morbidities associated with COVID-19 at an earlier stage.

## Introduction

COVID-19 infection is associated with a hypercoagulable state similar to various cytokine release syndromes. Several cases of vascular thrombosis with pulmonary embolism and mesenteric and portal vein thrombosis have been documented [1]. Most of these findings have been reported in critically ill patients with elevated acute phase reactants and deranged coagulation profiles [2-4]. Increased vasoconstrictors and inflammatory cytokines cause activation of coagulation pathways and the formation of thrombi.

## Case presentation

A 62-year-old female presented with right upper quadrant pain and loss of appetite over the prior 14 days. She gave no history of sore throat, dry cough, loss of taste, or fever; no other abdominal complaints were noted. On physical examination, the abdomen was soft, and there was no guarding, rigidity, or tenderness. No obvious organomegaly or mass was noted on palpation, and bowel sounds were normal. Electrocardiogram (ECG) and vitals were normal. For abdominal pain, an ultrasound (USG) of the abdomen was advised, which revealed dilation of the portal vein with a thrombus involving the main portal vein and its branches; Doppler ultrasound revealed no obvious blood flow within the portal vein. No splenomegaly or ascites was noted.

Liver function tests were within normal limits. The coagulation profile was normal. Increased levels of lactate dehydrogenase (346 U/L) were present, the total and differential leukocyte counts were within normal limits. Hemoglobin was 13.1 g/dL and serum creatinine was 0.71 mg/dL.

**Figure 1:**
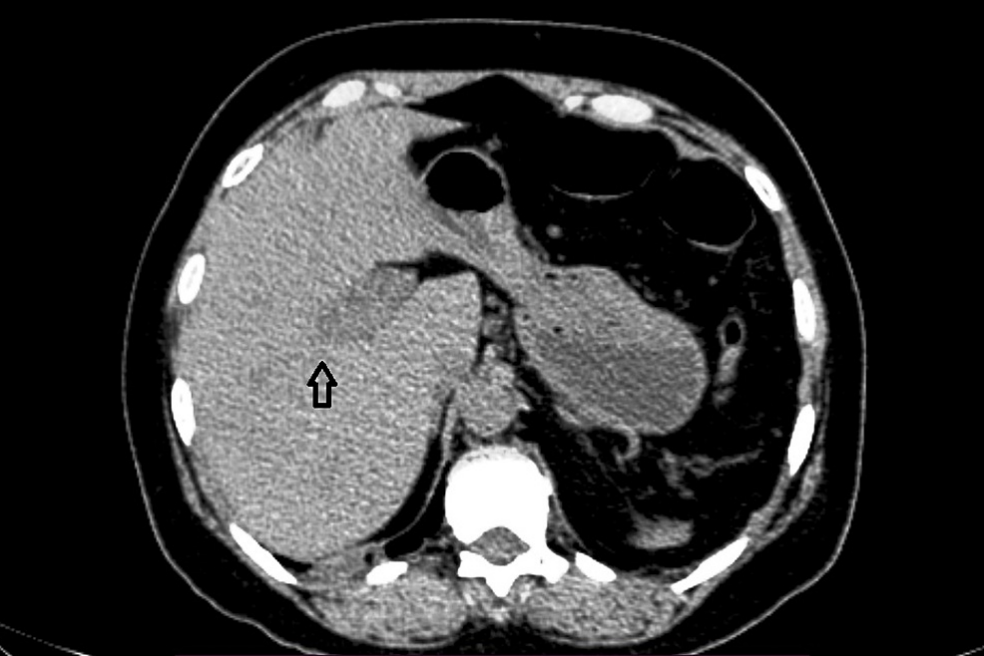
Non-contrast axial section showing the dilated hypodense portal vein (arrow).

**Figure 2:**
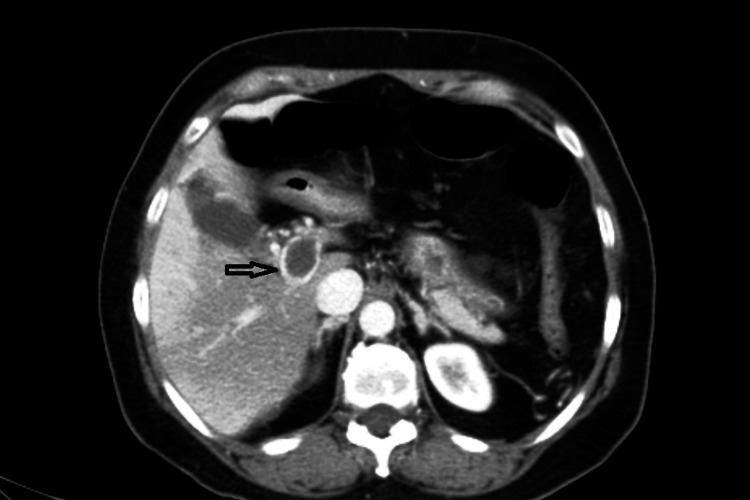
Post-contrast image showing a hypodense filling defect in the main portal vein (arrow).

**Figure 3:**
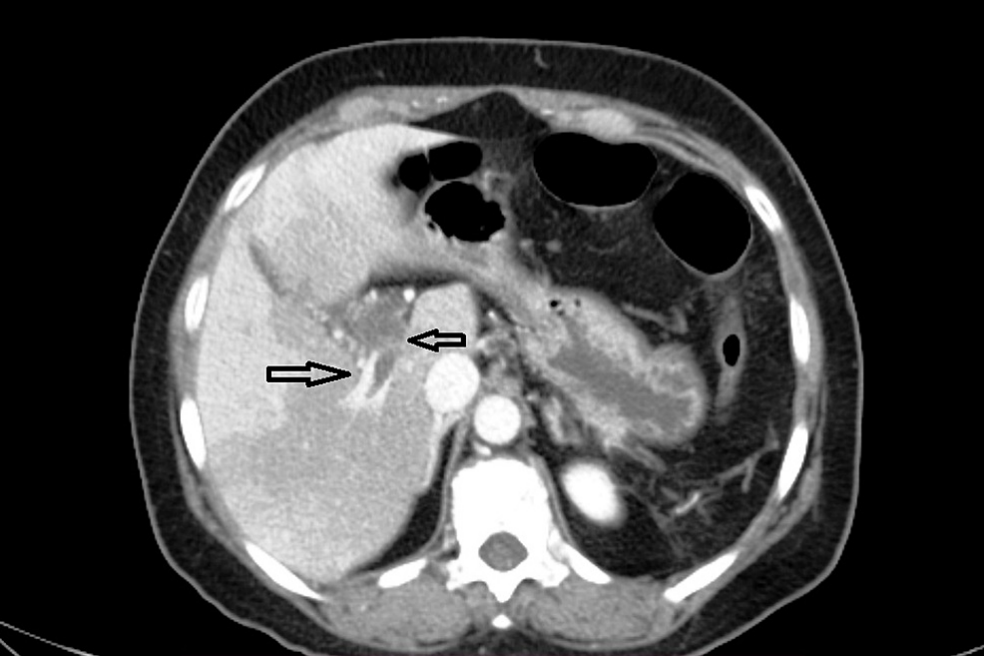
Axial image showing extension of a thrombus into the right branch of the portal vein and a few periportal collaterals (arrows).

**Figure 4:**
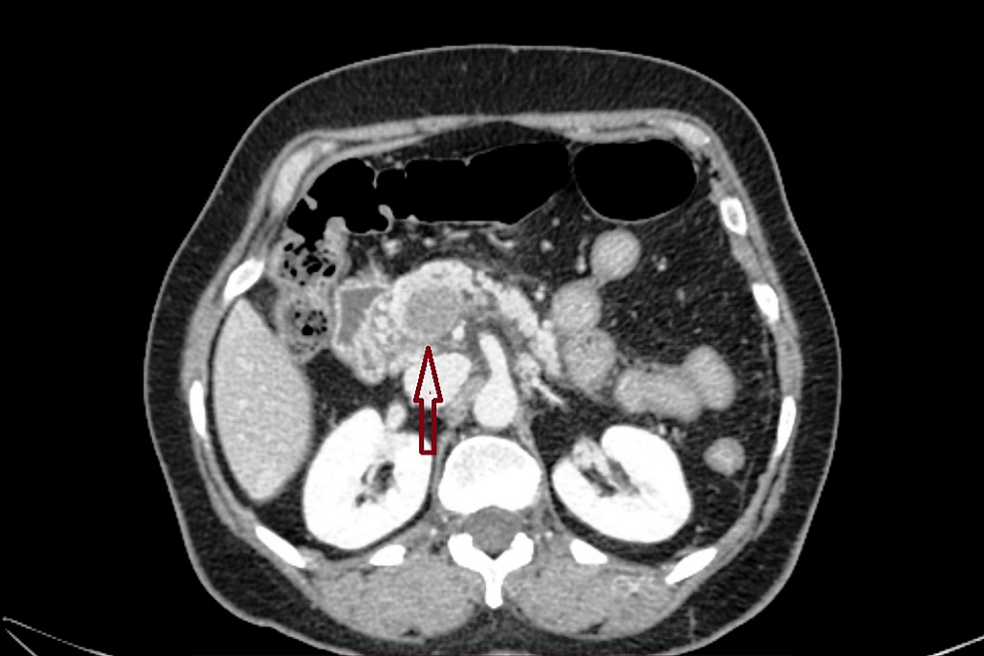
Post-contrast axial section at the level of the superior mesenteric vein shows the dilated superior mesenteric vein with a hypodense thrombus (arrow).

**Figure 5:**
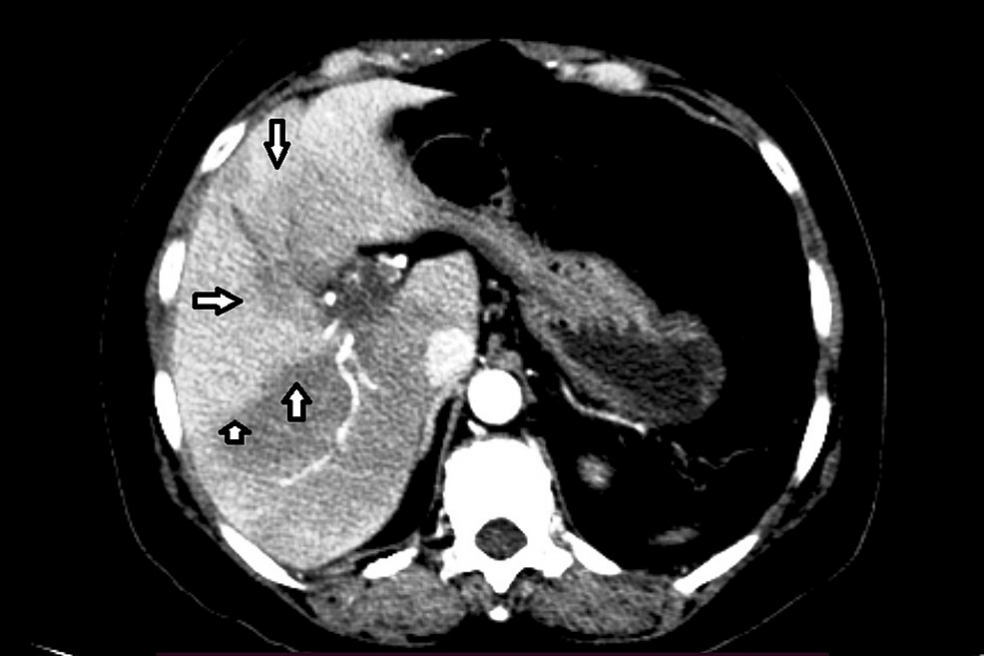
Arterial phase image showing the geographical area of difference in enhancement, which is not seen in the subsequent phase and is suggestive of transient hepatic attenuation difference (arrows).

**Figure 6:**
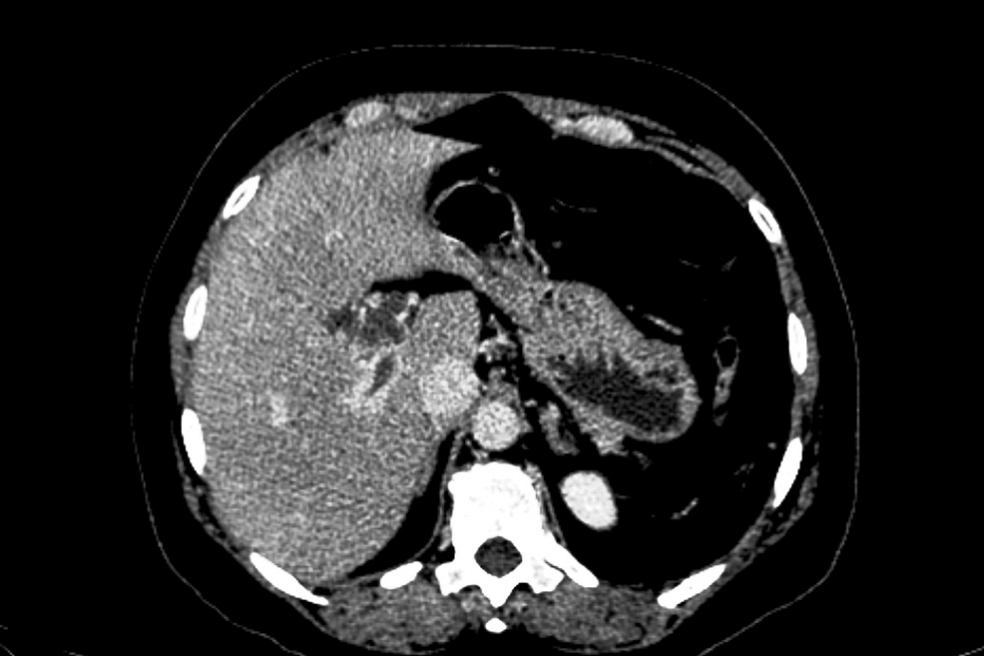
Homogenous enhancement of the liver is noted, consistent with the transient hepatic attenuation difference noted in the arterial phase in Figure 5.

Anticoagulation therapy was initiated with subcutaneous fondaparinux injection, 2.5 mg stat then once daily, and the patient was put on symptomatic treatment along with spironolactone, 25 mg once daily. Gastroenterology consultation suggested confirming USG findings with tri-phasic abdominal CT. CT findings suggested one large thrombus involving the superior mesenteric vein, the main portal vein with extension into its branches, and a few periportal collaterals. The liver and spleen were normal. No ascitic fluid was noted, and the rest of the abdominal organs were normal (Figures 1-6).

**Figure 7:**
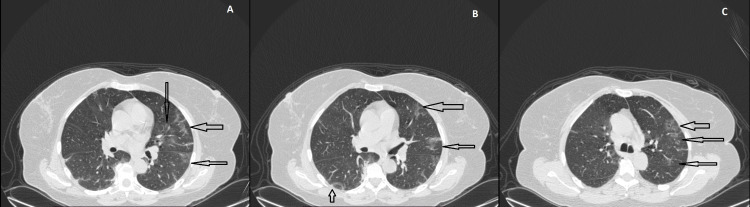
Patchy peripheral ground-glass opacities (arrows), suggestive of COVID-19, at different levels in 7A, 7B, and 7C.

After the CT findings suggested a thrombus in the portal vein and superior mesenteric vein with no history of an underlying coagulation disorder or cirrhosis, and keeping the current scenario of COVID-19 disease in mind, the suspicion of an underlying COVID-19 infection was raised and the patient was given SARS-CoV-2 total antibody (electrochemiluminescence immunoassay analyzer; ECLIA) test, which revealed a strongly positive result with 85.67 cut-off-index (COI) (>1 is reactive). Subsequently, the patient was given a high-resolution computed tomographic scan (HRCT) of the chest, which revealed multiple bilateral multisegmental peripheral ground-glass opacities consistent with COVID-19 infection. Figures 7A-7C are axial lung window HRCT images that show the above-mentioned changes.

A diagnosis of COVID-19-induced hypercoagulability leading to portal vein thrombosis was made and conservative medical management was advised. The patient was discharged from the hospital with a prescription for four more days of fondaparinux and acetrom (warfarin) at an initial dose of 4 mg (to be titrated according to the international normalized ratio; INR). Acetrom will be continued for at least 6 months, and regular INR testing and dose adjustments will be done on an outpatient basis.

## Discussion

Our patient, a 62-year-old female, presented with right upper quadrant pain and was subsequently diagnosed with a thrombus in the portal vein and superior mesenteric vein, without any bowel ischemia, due to deranged coagulation from an underlying COVID-19 infection.

Coronavirus leads to activation of the immune system and production of cytokines. The virus also interacts with the endothelial cells of the lung parenchyma via angiotensin-converting enzyme 2 (ACE2) receptors, leading to endothelial dysfunction and increased von Willebrand factor production, which, along with factor VIII release and tissue factor activation, results in abnormal coagulation and thrombus formation [5-9].

Most documented cases have shown pulmonary thromboembolism, ischemic stroke, acral ischemia, and mesenteric ischemia in patients with severe COVID-19. However, in our case, we found a large thrombus involving the superior mesenteric vein and the portal vein in a patient with subclinical COVID-19 infection, which is relatively a rare finding. A few documented cases have shown similar findings, but only in severely ill ICU patients. 

## Conclusions

The COVID-19 pandemic has been around for over a year now and has affected the entire world, with over 122 million cases and 2.7 million deaths. Various vascular complications have been noted due to SARS-CoV-2 infection, commonly, pulmonary thromboembolism, ischemic strokes, and mesenteric ischemia. Our case presents a rarer complication of subclinical COVID-19 causing thrombosis of the portal vein and superior mesenteric vein. Future studies will help further assess the risk of such vascular complications.

Studies are also required to correlate vascular complications with the severity of this disease. In the current scenario, any vascular complication without an underlying cause should raise suspicion of an underlying COVID-19 infection. 
